# Association between parental age at childbirth and timing of puberty in children: an 8-year cohort study

**DOI:** 10.1186/s13293-026-00877-x

**Published:** 2026-03-18

**Authors:** Qin Zhang, Chengpeng Cui, Zeqin Peng, Siyan Ye, Yan Li, Di Wu, Qin Liu

**Affiliations:** 1 Research Center for Environment and Human Health, Research Center for Medicine and Social Development, School of Public Health, Chongqing Medical University, Chongqing, China; 2 School of Nursing, Chongqing Medical University, Chongqing, China; 3 No.1 Yixueyuan Road, Yuzhong District, Chongqing, 400016 China

**Keywords:** Paternal age, Maternal age, Children, Puberty, Sex differences

## Abstract

**Background:**

The timing of puberty in offspring is influenced by various factors, including genetics and environment. While some studies suggest a link between parental age at childbirth and pubertal timing, the evidence remains inconsistent. This study aimed to investigate the association between parental age at reproduction and the timing of pubertal development events in their sons and daughters.

**Methods:**

We conducted a prospective cohort study with 1,386 children (51.7% girls) followed for a median of 7.00 years. Parental age was collected retrospectively. Pubertal development, including genital, breast, and pubic hair growth, as well as spermarche and menarche, was assessed semiannually. Accelerated Failure Time-Restrictive Cubic Spline models were used to analyze the impact of paternal and maternal age on the timing of these pubertal events.

**Results:**

Advanced maternal age at childbirth was significantly associated with an earlier age at spermarche in boys (*P* < 0.05). A non-linear association was also observed between maternal age at childbirth and the timing of genital development (*P* = 0.01). Both maternal and paternal ages at childbirth were significantly associated with the timing of breast and axillary hair development onset in girls in a non-linear, inverse U-shaped manner (*P* = 0.02 and *P* < 0.01, respectively), indicating that both younger and older parental ages are linked to the earlier breast and axillary hair development.

**Conclusions:**

This study suggests that parental age at childbirth is associated with pubertal development in offspring, including the timing of spermarche and genital development in boys and the onset of breast and axillary hair development in girls. Specifically, both younger and older parental ages have been linked to earlier attainment of these pubertal milestones, particularly among girls. In summary, the study showed that the specific pubertal events affected by parental age and the nature of that association were different for boys and girls.

**Plain english summary:**

The timing of puberty can affect a child’s future health. The aim of this study was to investigate whether a parent’s age at the time of childbirth influences when their child enters puberty. We conducted a long-term (with a median duration of 7 years) follow-up study on 1386 children to track their pubertal development. We found that the effects of parental age differ between boys and girls. For boys, having an older mother at birth was linked to reaching spermarche (first ejaculation) at a younger age. For girls, the findings were more complex. Both younger and older parental ages were linked to earlier breast and underarm hair development. This U-shaped pattern means that girls born to very young or very old parents tended to show these signs of puberty sooner than those born to parents in the middle-age range. In short, this study suggests that a parent’s age does play a role in the timing of their child’s puberty, but the effects are different for boys and girls. This information can help parents and doctors better understand the factors influencing child development.

**Highlights:**

1. Advanced Maternal Age and Earlier Puberty in Sons: Older maternal age at childbirth was significantly linked to boys reaching spermarche (first ejaculation) at a younger age
2. A U-Shaped Association for Daughters: For girls, both younger and older parental ages at childbirth were associated with an earlier onset of breast and underarm hair development
3. Gender-Specific Effects: The study highlights that the impact of parental age on puberty is different for boys and girls, affecting distinct pubertal milestones in a non-uniform pattern

**Supplementary Information:**

The online version contains supplementary material available at 10.1186/s13293-026-00877-x.

## Introduction

Puberty is a critical period marking the transition from childhood to adulthood, characterized by rapid physical growth, the development of secondary sexual characteristics, and the transformation of social roles [1]. Early and delayed timing of puberty can adversely affect a child’s physical and mental health [2]. Early pubertal timing is associated with an increased risk of future metabolic disorders and is directly linked to the incidence of hormone-related tumors [3, 4]. Moreover, early puberty timing is also correlated with a higher risk of psychological issues, early sexual debut, and sexual violence [5, 6]. Similarly, delayed puberty is associated with psychological challenges and compromised bone health in adulthood [2].

Global secular trends indicate a decline in the age at pubertal timing, characterized by breast development, among girls over recent decades [7]. Nutritional status is a well-established determinant of pubertal timing, with weight status serving as a primary influence on pubertal timing [4, 8]. In boys, long-term trends in pubertal timing and their association with nutritional status remain unclear [9], although limited evidence suggests a potential trend toward earlier timing [10]. Beyond nutritional factors, genetic and environmental influences also contribute to pubertal timing [10, 11]. Parental pubertal timing significantly impacts offspring development [12, 13], specifically, earlier maternal age at menarche is associated with earlier pubertal timing in both sons and daughters [13]. Furthermore, maternal conditions during pregnancy—such as obesity and exposure to stressful events—are linked to earlier pubertal development in offspring, with stress exhibiting a dose-dependent relationship [14, 15].

Childbearing age serves as a crucial indicator of reproductive health [16, 17]. Globally, the childbearing age is delaying [18, 19]. Parental age at childbirth is significantly associated with the health and developmental outcomes of their offspring. Advanced paternal age is associated with epigenetic alterations in sperm, which are transmitted to offspring [20], potentially elevating the risk of various adverse health outcomes in the next generation [21, 22]. Maternal age is associated with oocyte quality [17]. Advanced maternal age is linked to an increased risk of aneuploidy and genetic disorders in offspring [23], as well as reduced gestational age and birth weight [24, 25].

Prior research has indicated that younger maternal age has been associated with a lower risk of early menarche [26, 27]. However, conflicting findings exist, with some studies demonstrating no significant correlation between parental age and offspring pubertal timing [28, 29]. Consequently, further investigation is warranted to elucidate the precise impact of parental age on the pubertal development of offspring during adolescence.

Therefore, this study utilized an 8-year prospective cohort design involving semiannual physical examinations. We retrospectively collected data regarding parental age at childbirth and analyzed its association with the timing of key pubertal development events in offspring. This study aims to elucidate the influence of parental age at childbirth on the onset of pubertal milestones.

## Materials and methods

### Participants

Participants were drawn from a puberty development cohort in Chongqing, southwest China [30]. The baseline encompassed students in grades 1–4 across four primary schools. Eligibility criteria included enrollment in the specified grades and the absence of any medical conditions known to affect growth and development. All children participating in this study were Chinese. Of the 1429 children initially recruited, with a mean age of 8.59 years (SD = 1.20), forty-three children lacked data on either of their parents’ age at childbirth. Consequently, the final sample comprised 1386 participants. The cohort underwent biannual pubertal development assessments, commencing in April 2014 and concluding in May 2022, yielding a total of 17 repeated physical examination data.

### Questionnaires

A structured questionnaire was employed to gather data on participants’ birth dates, gender, monthly income per capita, parental education level, and feeding patterns before 6months. Parental age (in years) at childbirth, maternal age at menarche (in years) and participant birth weight (in grams) were documented through open-ended questionnaire items. Guardians of the participants completed all questionnaires, providing information based on reliable historical records or their recollection.

### Pubertal development assessments

Genitals, pubic hair, and axillary hair development in boys was assessed by a male investigator using the Tanner staging criteria [31], with testicular volume measured using a Prader orchidometer. In girls, breast development, pubic hair, and axillary hair development were evaluated by a female investigator using the Tanner staging criteria [32]. In instances of asymmetry, the more advanced stage was documented. Information regarding spermarche in boys and menarche in girls was collected through self-report during each survey. Participants were asked whether they had experienced these events since the previous follow-up, and if so, the month and year of their occurrence. Prior to the examination, investigators provided guidance on how to determine whether these events had taken place. To assist with recall, participants were prompted to identify memorable events such as festivals, class schedules, or holidays if they were unable to remember the exact month and year.

### Quality control

Pubertal development was assessed by fixed, separate, gender-specific investigators. Due to potential investigator turnover across follow-up surveys, rigorous measures were implemented to minimize measurement discrepancies. New investigators received training from outgoing investigators and were required to co-conduct physical examinations during a follow-up survey prior to assuming independent responsibility. A minimum of 90% inter-measurement consistency between new and outgoing investigators was mandated before independent data collection was permitted [33].

### Statistical analysis

EpiData 3.0 was utilized for data entry, with double entry verification employed to ensure accuracy. Maternal age at menarche was categorized into two groups: ≤13 years and > 13 years. Birth weight was classified into three categories: 2500–4000 g (normal birth weight), < 2500 g (low birth weight), and > 4000 g (macrosomia). Categorical variables were expressed as frequencies and percentages (N, %), while continuous variables were presented as means (SD/SEM/95% CI).

**Fig. 1:**
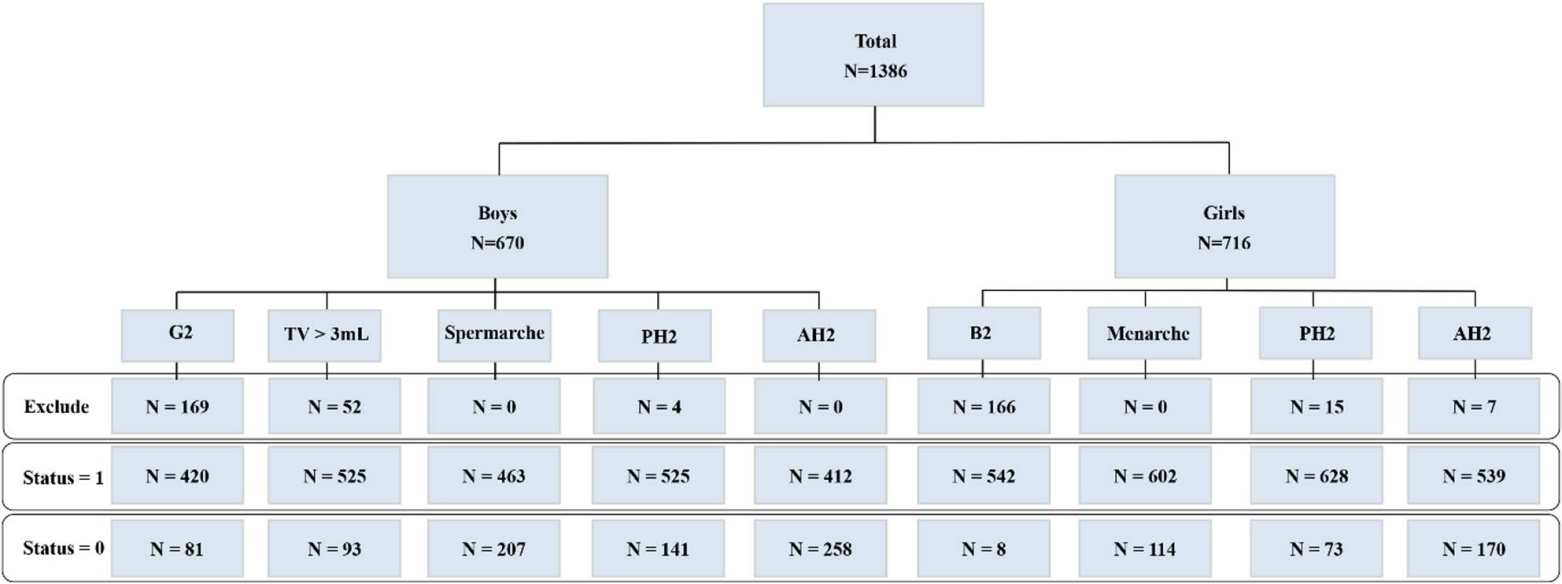
Analysis framework of the study. Exclude, those who had experienced pubertal events before recruitment and whose data were not observed were excluded. Status = 1, individuals who observed the onset of pubertal events and records their age. Status = 0, individuals who did not experience the onset of pubertal events during the follow-up period

The Accelerated Failure Time (AFT) model, using a Weibull distribution, was employed to analyze the relationship between the age of childbearing and the timing of pubertal events. The Restrictive Cubic Spline (RCS) was used to reflect the nonlinear relationship between the age of childbearing and the Time Ratio (TR). Model selection was guided by the Akaike Information Criterion (AIC) as the primary metric and the Bayesian Information Criterion (BIC) (A lower AIC value indicates better model fit. Consequently, the knots associated with the lowest AIC value were selected. Supplementary Table S1). The outcome events, marking pubertal milestones, were defined as follows: for boys, the attainment of genital Tanner stage 2 (G2), testicular volume exceeding 3 mL (TV > 3 mL), occurrence of spermarche, and the attainment of pubic hair Tanner stage 2 (PH2) and axillary hair Tanner stage 2 (AH2); for girls, the attainment of breast Tanner stage 2 (B2), occurrence of menarche, and the attainment of PH2 and AH2. Children who had experienced pubertal events prior to cohort entry and were unable to recall the specific timing were excluded from the analysis. Survival time (Timing of pubertal events) was defined as the age at the midpoint between the visit where the event was first observed and the immediately preceding visit, or as the self-reported age at the time of spermarche or menarche. Children who had not experienced the endpoint event were retained in the dataset, with their last observation time (censoring time) and censoring status recorded. Paternal age at childbirth, maternal age at childbirth, monthly income per capita, father’s education level, mother’s education level, and maternal age at menarche were included in the final model. Figure 1 illustrates the sample size included in the final analysis for each pubertal event. Characteristics between completers and non-completers of each pubertal indicator were compared in Supplementary Table S2. All analyses were conducted using R version 4.4.1. The “*survival*” and “*rms*” package were utilized for AFT and RCS models. A two-tailed p-value of less than 0.05 was considered statistically significant. Due to forgetting, omission and refusal, some covariates are missing. We interpolated the missing values using the multiple interpolation and conducted the sensitivity analysis.

## Results

### Basic information of the subjects

A total of 1,386 children were included in the study. The mean age at baseline was 8.58 years, with 51.7% being girls. The average paternal age at childbirth was 29.57 years (median = 29, range: 19–55), and the average maternal age at childbirth was 27.05 years (median = 26, range: 17–49), with 95% of fathers aged 21 to 40 years and 95% of mothers aged 20 to 38 years. (Table 1 and Supplementary Figure S1). The average age at G2, TV > 3 mL, spermarche, PH2, and AH2 in boys was 11.82, 11.82, 12.82, 12.52, and 13.70 years, respectively. The mean age at B2, menarche, PH2, and AH2 in girls was 10.18, 11.87, 11.60, and 12.47 years, respectively (Table 2).

**Table 1:** Characteristics of the participants

Characteristics	Boys, *n* = 670 N (%)	Girls, *n* = 716 N (%)	Total, *n* = 1386 N (%)
Age^*^, year	8.58(1.19)	8.59(1.21)	8.58(1.20)
Monthly income per capita			
≤ 2000 RMB	203(30.3)	219(30.6)	422(30.5)
> 2000 RMB	467(69.7)	496(69.4)	963(69.5)
Father’s education level			
Primary and junior high schools	304(45.5)	328(45.8)	632(45.7)
Senior high school	232(34.7)	230(32.1)	462(33.4)
College or above	132(19.8)	158(22.1)	290(21.0)
Mother’s education level			
Primary and junior high schools	328(49.2)	351(49.1)	679(49.1)
Senior high school	221(33.1)	227(31.7)	448(32.4)
College or above	118(17.7)	137(19.2)	255(18.5)
Feeding patterns before 6months			
Exclusive breastfeeding	436(65.1)	471(65.9)	907(65.5)
Mixed feeding	119(17.8)	128(17.9)	247(17.8)
Formula	115(17.2)	116(16.2)	231(16.7)
Birth weight			
2500–4000 g	590(88.6)	641(90.0)	1231(89.3)
< 2500 g	22(3.3)	34(4.8)	56(4.1)
> 4000 g	54(8.1)	37(5.2)	91(6.6)
Maternal age at menarche			
≤ 13years	295(45.0)	364(51.4)	659(48.3)
> 13years	361(55.0)	344(48.6)	705(51.7)
Paternal age at childbirth^*^, year	29.49(4.99)	29.65(5.22)	29.57(5.11)
Maternal age at childbirth^*^, year	27.03(4.90)	27.07(5.04)	27.05(4.97)

*Mean (SD)

**Table 2:** Age at study endpoints of pubertal events, year

	Boys, Mean ± SD			Girls, Mean ± SD	
	Status = 1	Status = 0		Status = 1	Status = 0
G2	11.82 ± 1.37	11.93 ± 0.88		-	-
TV > 3 mL	11.82 ± 1.16	12.11 ± 1.01		-	-
First spermatorrhea	12.82 ± 1.27	12.89 ± 1.53		-	-
B2	-	-		10.18 ± 0.94	11.20 ± 1.44
Menarche	-	-		11.87 ± 0.94	12.01 ± 1.19
PH2	12.52 ± 1.02	12.14 ± 0.93		11.60 ± 0.85	11.72 ± 0.82
AH2	13.70 ± 1.06	12.85 ± 1.42		12.47 ± 1.17	12.40 ± 1.52

Status = 1, participants who had reached the specific pubertal marker (event occurred), and the data represent survival time or timing of pubertal events. Status = 0, participants who were censored (had not yet reached the marker by the time of assessment), and the data represent censoring time

### Effects of parents’ age at childbirth on pubertal timing in boys

**Fig. 2:**
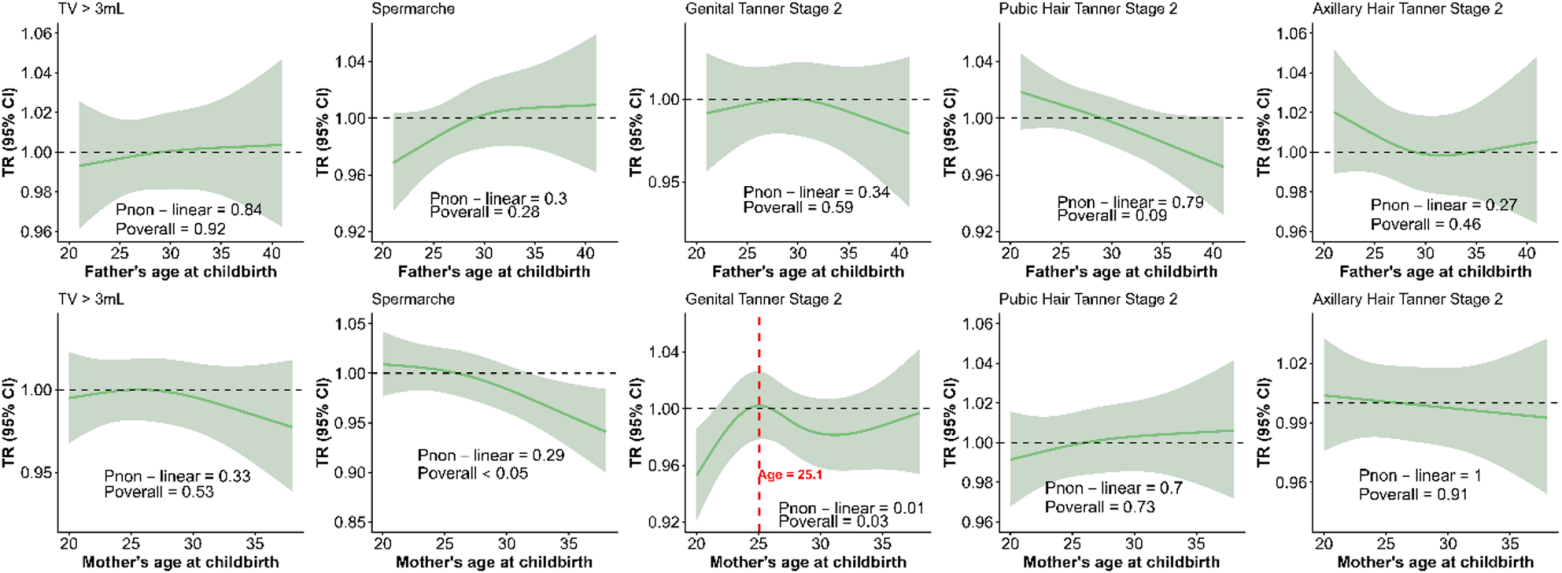
Association between parents’ age at childbirth and the timing of puberty in boys. The TR represents the effect of a predictor variable on the time scale. The median age of childbearing was set as the reference (TR = 1). The solid green line represents the average effect curve predicted by the model. The light green shading indicates the 95% confidence interval (CI). A TR greater than 1 indicates a delay in pubertal timing, while a value less than 1 indicates earlier onset. The red vertical dashed line and corresponding values indicate a significant nonlinear correlation, marking the specific parental age at which the direction of the association with the TR of pubertal events onset in boys shifts, coinciding with the peak TR value

No significant association was observed between paternal age at childbirth and the onset of puberty or puberty-related events in boys. Advanced maternal age at childbirth was significantly associated with an earlier age at spermarche in boys (*P* < 0.05). A significant non-linear association was observed between maternal age at childbearing and the timing of external genital development in boys (*P* = 0.01); and the overall correlation was significant (*P* = 0.03) (Fig. 2). The correlation between maternal age at childbirth and the timing of external genital development in boys was potentially significant upon sensitivity analysis (Supplementary Figure S2).

### Effects of parents’ age at childbirth on children’s pubertal timing in girls

**Fig. 3:**
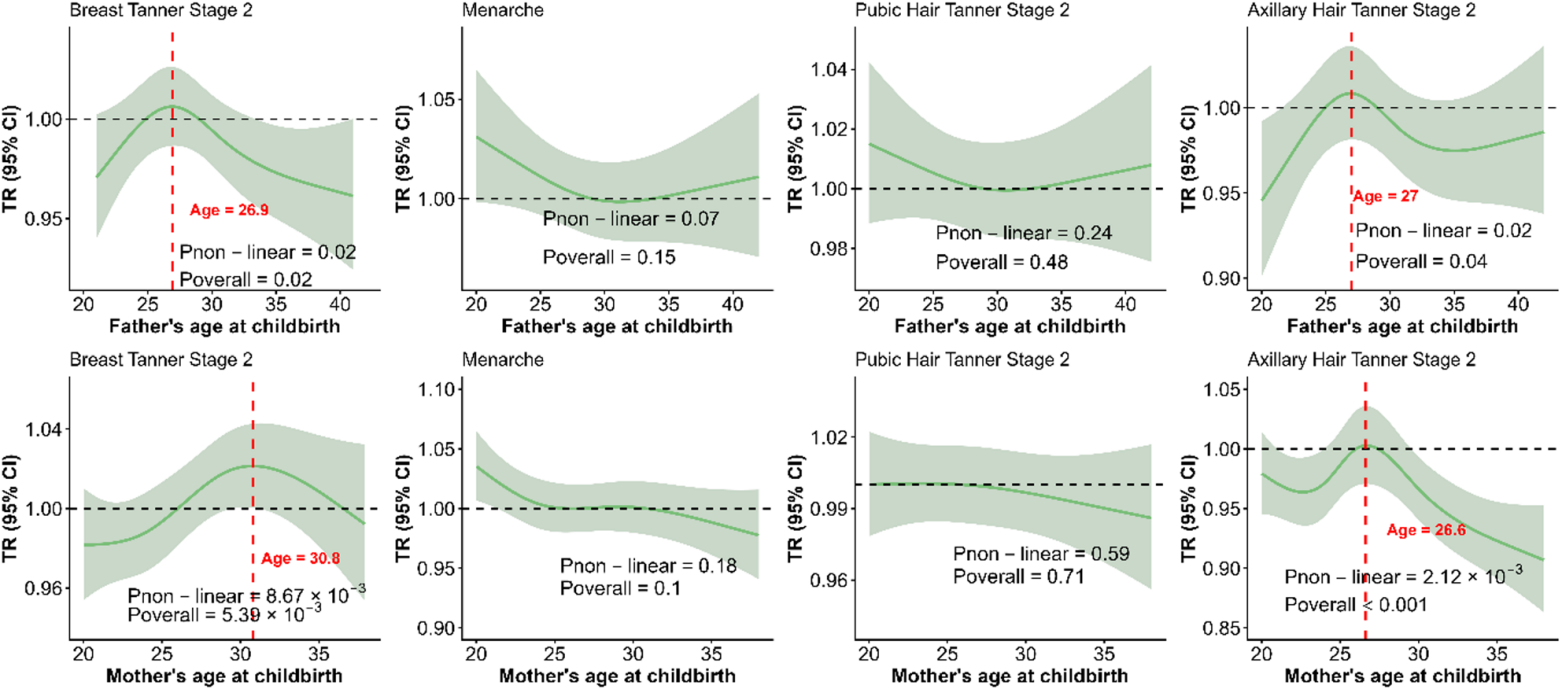
Association between parents’ age at childbirth and the timing of puberty in girls. The TR represents the effect of a predictor variable on the time scale. The median age of childbearing was set as the reference (TR = 1). The solid green line represents the average effect curve predicted by the model. The light green shading indicates the 95% CI. A TR greater than 1 indicates a delay in pubertal timing, while a value less than 1 indicates earlier onset. The red vertical dashed line and corresponding values indicate a significant nonlinear correlation, marking the specific parental age at which the direction of the association with the TR of pubertal events onset in girls shifts, coinciding with the peak TR value

Both paternal and maternal age at childbirth exhibited significant non-linear, inverse U-shaped associations with the timing of breast (*P* = 0.02 and *P* < 0.01, respectively) and axillary hair (*P* = 0.02 and *P* < 0.01, respectively) development onset in girls, indicating that both younger and older parental ages at childbirth might be linked to earlier onset of breast and axillary hair development (Fig. 3). Through sensitivity analysis, it was found that the results were stable (Supplementary Figure S3).

## Discussion

This study utilized longitudinal cohort data to investigate the non-linear association between parental age at childbirth and the timing of pubertal milestones in offspring. The findings suggest that the relationship is not linear in most cases; rather, both younger and older parental ages may be associated with earlier pubertal development in their children, particularly among girls.

Consistent with some prior research [26, 27], our study observed a non-significant or potential trend toward delayed menarche in daughters of younger mothers. In contrast, we found that younger maternal age was associated with earlier breast development in daughters. Previously, evidence was lacking for an association between advanced paternal age and early breast development in daughters, as well as between advanced maternal age and earlier spermarche in sons and earlier axillary hair development daughters. Limited evidence points to a positive association between paternal age and offspring breast cancer risk, an association that is further strengthened when controlling for maternal age [34, 35]. Research indicates a potential U-shaped association between maternal age and testicular volume in young man, with follicle-stimulating hormone (FSH) and luteinizing hormone (LH) levels tending to increase as maternal age advances [36]. Daughters of older mothers may exhibit more masculine traits [37]. Conversely, some studies have reported elevated testosterone levels in the amniotic fluid and female fetuses of younger mothers [38, 39]. And prenatal exposure to elevated androgen levels may accelerate adrenal development in female offspring [40]. Furthermore, paternal age at conception may also be linked to adrenal hormone levels—specifically dehydroepiandrosterone (DHEA) and testosterone (T)—in children aged 6–8 years [41].

The mechanisms linking parental age to offspring pubertal development are complex and remain incompletely understood. Parental effects are defined as the causal influence of parental genotype and phenotype on offspring phenotype [42, 43]. As previously mentioned, age-related changes in germ cell quality and reproductive hormones likely play a role [17, 44, 45]. Besides, a substantial body of evidence indicates that aging alters the genetic material content in germ cells, thereby affecting the health of future generations [20, 46–48]. Epidemiological evidence suggests that parental age may be related to daughters’ physical growth and affect their metabolic function [49, 50]. Animal studies have provided insights into potential pathways; for instance, advanced paternal age in mice has been linked to altered metabolic signaling and accelerated aging in offspring [51, 52], while other models suggest energy metabolism pathways may be involved [53]. However, given that biological markers were not directly measured in the present study, these explanations remain speculative. We hypothesize that parental age may influence growth and biological aging by modulating energy metabolism, thereby indirectly affecting pubertal timing, but this requires validation in future mechanistic studies.

Resources and economic conditions can significantly influence the growth and development of children and adolescents (Supplementary Table S3) [54]. Simultaneously, socioeconomic factors influence childbearing timing (Supplementary Table S3); individuals with limited resources tend to have children earlier, whereas those with higher educational attainment often delay parenthood or have lower fertility intentions [55, 56]. However, economic pressures have also been associated with the postponement of childbearing plans [57]. Furthermore, among women of childbearing age, socioeconomic status may indirectly influence ovarian reserve through pathways involving nutrition, stress, and environmental factors [58]. Childbearing age may influence birth mode, indirectly impacting pubertal timing. Advanced maternal age is a well-established risk factor for cesarean Sects [59, 60]. Cesarean section has been linked to an earlier timing of puberty in males and may influence growth trajectories in preschool children [61, 62]. Thus, the complex pubertal development outcomes observed in this study likely result from a multifactorial interplay. Consequently, variations in pubertal development among children of different parental age at childbirth may result from a combination of parental genetic and environmental factors.

This study employed an eight-year cohort design, with physical examinations of children and adolescents conducted by professionals every six months. This approach allows for more accurate observation and documentation of adolescent growth and development compared to self-reporting and longer assessment intervals. Nevertheless, the study has several limitations. First, the sample size was relatively small. Second, participants were still undergoing growth, and no final developmental state was observed, with a 5-year loss to follow-up rate of 28.2%. The primary cause of attrition was the transition of students from junior high to high school, which disrupted the school-based follow-up. Consequently, data for individuals with later pubertal maturation or for events typically occurring at older ages may be disproportionately missing. This selective censoring could lead to an underestimation of the age at later pubertal milestones, and differences in baseline characteristics between completers and non-completers for each pubertal indicator may affect the stability of the results. Third, numerous factors influence pubertal development, and the study lacked sufficient control for covariates, such as parental weight status and other pre-pregnancy and gestational conditions. Fourth, the range of parental age at childbirth was narrow, without observations on the effects of extreme age values, the study could not assess whether minimal and maximal childbearing age might have more significant or different adverse effects on offspring health [63–65]. Therefore, caution is warranted when extrapolating the current findings to very young or advanced parental ages, as the associations observed in this cohort may not fully apply to these specific subgroups. And a correlation exists between paternal and maternal age at childbirth (Supplementary Figure S1), collectively influencing offspring development. Therefore, analyzing their interactive effects may provide more meaningful insights into their impact on children’s pubertal development. In addition, although parental age is generally considered a reliable variable due to its objective nature and high salience to guardians, the reliance on self-report introduces the possibility of measurement error. However, we expect such errors to be minimal and non-differential with respect to the offspring’s pubertal outcomes. Finally, the analysis of multiple pubertal milestones increases the statistical risk of Type I error. Therefore, although the observed patterns are biologically plausible, the results should be interpreted cautiously. Our findings indicate that parental age is associated with shifts in pubertal timing. While this magnitude is modest and may have limited clinical consequences at the individual level, it remains meaningful from a population and biological perspective, especially in the context of changing global demographics, where delayed childbearing is becoming more common. These findings support the biological relevance of parental age in shaping developmental trajectories and may influence societal attitudes towards family planning and the timing of reproduction.

## Conclusions

Our findings suggest an association between parental age at childbirth and the timing of some pubertal milestones in offspring. Specifically, advanced maternal age was associated with an earlier age at spermarche in boys, and there is a non-linear correlation between maternal age and genital development. For girls, both younger and older parental ages were linked to earlier breast and axillary hair development, demonstrating a significant inverse U-shaped association. These findings elucidate the complex relationship between parental age and offspring pubertal development, with significant implications for public health, biological research, and social policy. However, the mechanisms underlying the observed sex-specific differences in this association remain unclear, underscoring the need for further investigation.

## Supplementary Information

Below is the link to the electronic supplementary material.

Supplementary Material 1

## Data Availability

Data for this study are available from the corresponding author upon reasonable request. By E-mail, [liuqin@cqmu.edu.cn](mailto: liuqin@cqmu.edu.cn) .
