# Supplementary material for: Association between parental age at childbirth and timing of puberty in children: an 8-year cohort study
Source: Biol Sex Differ. 2026 Mar 18;17:88. doi: 10.1186/s13293-026-00877-x (PMC13112619; doi:10.1186/s13293-026-00877-x)
Supplement: Supplementary file 1 — Supplementary Material 1 [file 13293_2026_877_MOESM1_ESM.docx]

**Contents**

[**Supplementary Table S1** Models fitting. 2](#_Toc223016906)

[**Supplementary Table S2** Comparison of characteristics between completers and non-completers of each pubertal indicator. 3](#_Toc223016907)

[**Supplementary Table S3** Results of spearman correlation analysis. 5](#_Toc223016908)

[**Supplementary Figure S1** Correlation between paternal and maternal age at childbirth. 6](#_Toc223016909)

[**Supplementary Figure S2** Association between parents' age at childbirth and the timing of puberty in boys (interpolated data). 7](#_Toc223016910)

[**Supplementary Figure S3** Association between parents' age at childbirth and the timing of puberty in girls (interpolated data). 8](#_Toc223016911)

**Supplementary Table S1** Models fitting.

|  | **Paternal Age** | | | | | | **Maternal Age** | | | | | |
| --- | --- | --- | --- | --- | --- | --- | --- | --- | --- | --- | --- | --- |
|  | Knots = 3 | | Knots = 4 | | Knots = 5 | | Knots = 3 | | Knots = 4 | | Knots = 5 | |
|  | AIC | BIC | AIC | BIC | AIC | BIC | AIC | BIC | AIC | BIC | AIC | BIC |
| **Boy** |  |  |  |  |  |  |  |  |  |  |  |  |
| TV > 3mL | 1841.8 | 1890.3 | 1843.2 | 1896.1 | 1843.8 | 1901.0 | 1840.9 | 1889.4 | 1841.7 | 1894.5 | 1843.9 | 1901.2 |
| Spermarche | 1948.6 | 1997.9 | 1950.5 | 2004.3 | 1951.5 | 2009.8 | 1948.5 | 1997.8 | 1950.5 | 2004.3 | 1952.2 | 2010.5 |
| Genital Tanner Stage 2 | 1532.9 | 1579.0 | 1534.6 | 1584.9 | 1535.8 | 1590.3 | 1531.3 | 1577.4 | 1527.6 | 1577.9 | 1529.6 | 1584.1 |
| Pubic Hair Tanner Stage 2 | 1703.1 | 1752.4 | 1705.1 | 1758.8 | 1705.8 | 1764.0 | 1703.1 | 1752.3 | 1703.8 | 1757.5 | 1705.9 | 1764.1 |
| Axillary Hair Tanner Stage 2 | 1548.6 | 1598.0 | 1550.4 | 1604.2 | 1551.8 | 1610.1 | 1549.9 | 1599.2 | 1551.1 | 1604.9 | 1553.0 | 1611.3 |
| **Girl** |  |  |  |  |  |  |  |  |  |  |  |  |
| Breast Tanner Stage 2 | 1548.5 | 1595.7 | 1547.2 | 1598.7 | 1547.4 | 1603.1 | 1547.7 | 1594.9 | 1545.9 | 1597.3 | 1545.9 | 1601.6 |
| Menarche | 2018.8 | 2068.9 | 2019.2 | 2073.9 | 2021.2 | 2080.4 | 2022.6 | 2072.6 | 2021.8 | 2076.5 | 2023.9 | 2083.1 |
| Pubic Hair Tanner Stage 2 | 1813.1 | 1862.9 | 1814.3 | 1868.7 | 1816.3 | 1875.2 | 1814.2 | 1864.1 | 1815.9 | 1870.3 | 1818.0 | 1876.9 |
| Axillary Hair Tanner Stage 2 | 2169.5 | 2219.5 | 2164.5 | 2219.1 | 2165.2 | 2224.2 | 2160.5 | 2210.5 | 2163.2 | 2217.7 | 2159.5 | 2218.5 |

Note. Knots are specific percentiles of the exposure variable distribution where the shape of the curve is allowed to change, enabling the model to capture non-linear relationships.

**Supplementary Table S2** Comparison of characteristics between completers and non-completers of each pubertal indicator.

|  |  | Paternal age at childbirth, Mean(SD) | Maternal age at childbirth, Mean(SD) | Monthly income per capita, N(%) | | Father's education level, N(%) | | | Mother's education level, N(%) | | | Maternal age at menarche, N(%) | |
| --- | --- | --- | --- | --- | --- | --- | --- | --- | --- | --- | --- | --- | --- |
|  |  |  |  | ≤2000 RMB | >2000 RMB | Primary and junior high schools | Senior high school | College or above | Primary and junior high schools | Senior high school | College or above | ≤13years | >13years |
| **Boy** |  |  |  |  |  |  |  |  |  |  |  |  |  |
| TV > 3mL | Non-completers | 29.5（4.8） | 26.9(4.6) | 20(21.5) | 73(78.5) | 31(33.3) | 37(39.8) | 25(26.9) | 41(44.6) | 29(31.5) | 22(23.9) | 37(40.7) | 54(59.3) |
|  | Completers | 29.5（5.0） | 27.1(4.9) | 165(31.4) | 360(68.6) | 249(47.4) | 177(33.7) | 99(18.9) | 262(50.1) | 171(32.7) | 90(17.2) | 226(44.0) | 288(56.0) |
|  | t/χ^2^ | -0.066 | -0.462 | 3.709 |  | 6.841* |  |  | 2.450 |  |  | 0.345 |  |
| Spermarche | Non-completers | 29.3(4.6) | 26.8(4.5) | 60(29.0) | 147(71.0) | 84(40.8) | 73(35.4) | 49(23.8) | 89(43.2) | 72(35.0) | 45(21.8) | 89(43.8) | 114(56.2) |
|  | Completers | 29.6(5.1) | 27.1(5.0) | 143(30.9) | 320(69.1) | 220(47.6) | 159(34.4) | 83(18.0) | 239(51.8) | 149(32.3) | 73(15.8) | 206(45.5) | 247(54.5) |
|  | t/χ^2^ | -0.628 | -0.714 | 0.245 |  | 3.952 |  |  | 5.365 |  |  | 0.151 |  |
| G2 | Non-completers | 29.4(4.7) | 27.2(4.7) | 18(22.2) | 63(77.8) | 24(29.6) | 33(40.7) | 24(29.6) | 32(39.5) | 28(34.6) | 21(25.9) | 36(44.4) | 45(55.6) |
|  | Completers | 29.7(5.2) | 27.1(5.0) | 130(31.0) | 290(69.0) | 202(48.1) | 142(33.8) | 76(18.1) | 215(51.6) | 128(30.7) | 74(17.7) | 179(43.9) | 229(56.1) |
|  | t/χ^2^ | -0.447 | 0.127 | 2.486 |  | 10.593* |  |  | 4.690 |  |  | 0.009 |  |
| PH2 | Non-completers | 29.3(4.9) | 27.2(4.9) | 36(25.5) | 105(74.5) | 50(35.7) | 56(40.0) | 34(24.3) | 64(45.4) | 46(32.6) | 31(22.0) | 60(43.2) | 79(56.8) |
|  | Completers | 29.5(5.0) | 27.0(4.9) | 166(31.6) | 359(68.4) | 254(48.5) | 173(33.0) | 97(18.5) | 263(50.4) | 172(33.0) | 87(16.7) | 234(45.6) | 279(54.4) |
|  | t/χ^2^ | -0.400 | 0.387 | 1.949 |  | 7.358* |  |  | 2.330 |  |  | 0.265 |  |
| AH2 | Non-completers | 29.2(4.8) | 26.8(4.7) | 72(27.9) | 186(72.1) | 103(40.1) | 93(36.2) | 61(23.7) | 116(45.0) | 81(31.4) | 61(23.6) | 109(42.9) | 145(57.1) |
|  | Completers | 29.7(5.1) | 27.2(5.0) | 131(31.8) | 281(68.2) | 201(48.9) | 139(33.8) | 71(17.3) | 212(51.8) | 140(34.2) | 57(13.9) | 186(46.3) | 216(53.7) |
|  | t/χ^2^ | -1.161 | -0.937 | 1.136 |  | 6.302* |  |  | 10.329* |  |  | 0.708 |  |
| **Girl** |  |  |  |  |  |  |  |  |  |  |  |  |  |
| B2 | Non-completers | 29.8(5.5) | 28.0(4.4) | 2(25.1) | 6(75.0) | 5(62.5) | 1(12.5) | 2(25.0) | 3(37.5) | 3(37.5) | 2(25.0) | 3(37.5) | 5(62.5) |
|  | Completers | 29.7(5.2) | 27.1(5.1) | 167(30.9) | 374(69.1) | 254(46.9) | 163(30.1) | 125(23.1) | 265(49.0) | 166(30.7) | 110(20.3) | 261(48.7) | 274(51.2) |
|  | t/χ^2^ | 0.046 | 0.505 | 1.000 ^#^ |  | 0.587 ^#^ |  |  | 0.718 ^#^ |  |  | 0.725 ^#^ |  |
| Menarche | Non-completers | 30.1(5.3) | 27.0(5.1) | 35(30.7) | 79(69.3) | 52(45.6) | 24(21.1) | 38(33.3) | 51(45.1) | 30(26.5) | 32(28.3) | 49(43.8) | 63(56.2) |
|  | Completers | 29.6(5.2) | 27.0(5.0) | 184(30.6) | 417(69.4) | 276(45.8) | 206(34.2) | 120(19.9) | 300(49.8) | 197(32.7) | 105(17.4) | 315(52.9) | 281(47.1) |
|  | t/χ^2^ | 1.032 | -0.267 | <0.001 |  | 12.973* |  |  | 7.445* |  |  | 3.127 |  |
| PH2 | Non-completers | 30.8(5.3) | 27.7(5.1) | 25(34.2) | 48(65.8) | 36(49.3) | 13(17.8) | 24(32.9) | 36(49.3) | 18(24.7) | 19(26.0) | 33(45.8) | 39(54.2) |
|  | Completers | 29.5(5.2) | 27.0(5.0) | 189(30.1) | 438(69.9) | 287(45.7) | 209(33.3) | 132(21.0) | 307(49.0) | 204(32.5) | 116(18.5) | 322(51.9) | 299(48.1) |
|  | t/χ^2^ | 2.048* | 1.214 | 0.519 |  | 9.261* |  |  | 3.202 |  |  | 0.935 |  |
| AH2 | Non-completers | 29.5(5.3) | 26.8(4.9) | 56(32.9) | 114(67.1) | 82(48.2) | 34(20.0) | 54(31.8) | 84(49.7) | 45(26.6) | 40(23.7) | 74(44.3) | 93(55.7) |
|  | Completers | 29.7(5.2) | 27.1(5.1) | 160(29.7) | 378(70.3) | 242(44.9) | 193(35.8) | 104(19.3) | 263(48.8) | 180(33.4) | 96(17.8) | 285(53.4) | 249(46.6) |
|  | t/χ^2^ | -0.451 | -0.707 | 0.624 |  | 19.419** |  |  | 4.175 |  |  | 4.179* |  |

Note. To compare continuous variables between groups, t-tests were employed, while categorical variables were analyzed using chi-square tests. *P<0.05, **P<0.001, ^#^ P value of Fisher's exact test.

**Supplementary Table S3** Results of spearman correlation analysis.

|  | Father's education level | Mother's education level | Monthly income per capita | Maternal age at menarche |
| --- | --- | --- | --- | --- |
| Boy |  |  |  |  |
| Paternal age at childbirth | -0.06 | -0.061 | -0.163** | 0.022 |
| Maternal age at childbirth | -0.104** | -0.099* | -0.182** | 0.054 |
| Age at TV>3mL | -0.094* | -0.134** | -0.101* | 0.026 |
| Age at first spermatorrhea | -0.088* | -0.078* | -0.093* | 0.038 |
| Age at G2 | -0.095* | -0.114* | -0.133** | 0.085 |
| Age at PH2 | -0.158** | -0.173** | -0.109** | 0.028 |
| Age at AH2 | -0.172** | -0.144** | -0.105** | -0.007 |
| Girl |  |  |  |  |
| Paternal age at childbirth | -0.075* | -0.132** | -0.167** | 0.092* |
| Maternal age at childbirth | -0.076* | -0.138** | -0.198** | 0.139** |
| Age at B2 | -0.108* | -0.067 | -0.024 | 0.130** |
| Age at menarche | -0.101** | -0.082* | -0.059 | 0.157** |
| Age at PH2 | -0.131** | -0.158** | -0.075* | 0.154** |
| Age at AH2 | -0.116** | -0.115** | -0.04 | 0.058 |

Note. *P<0.05, **P<0.01.


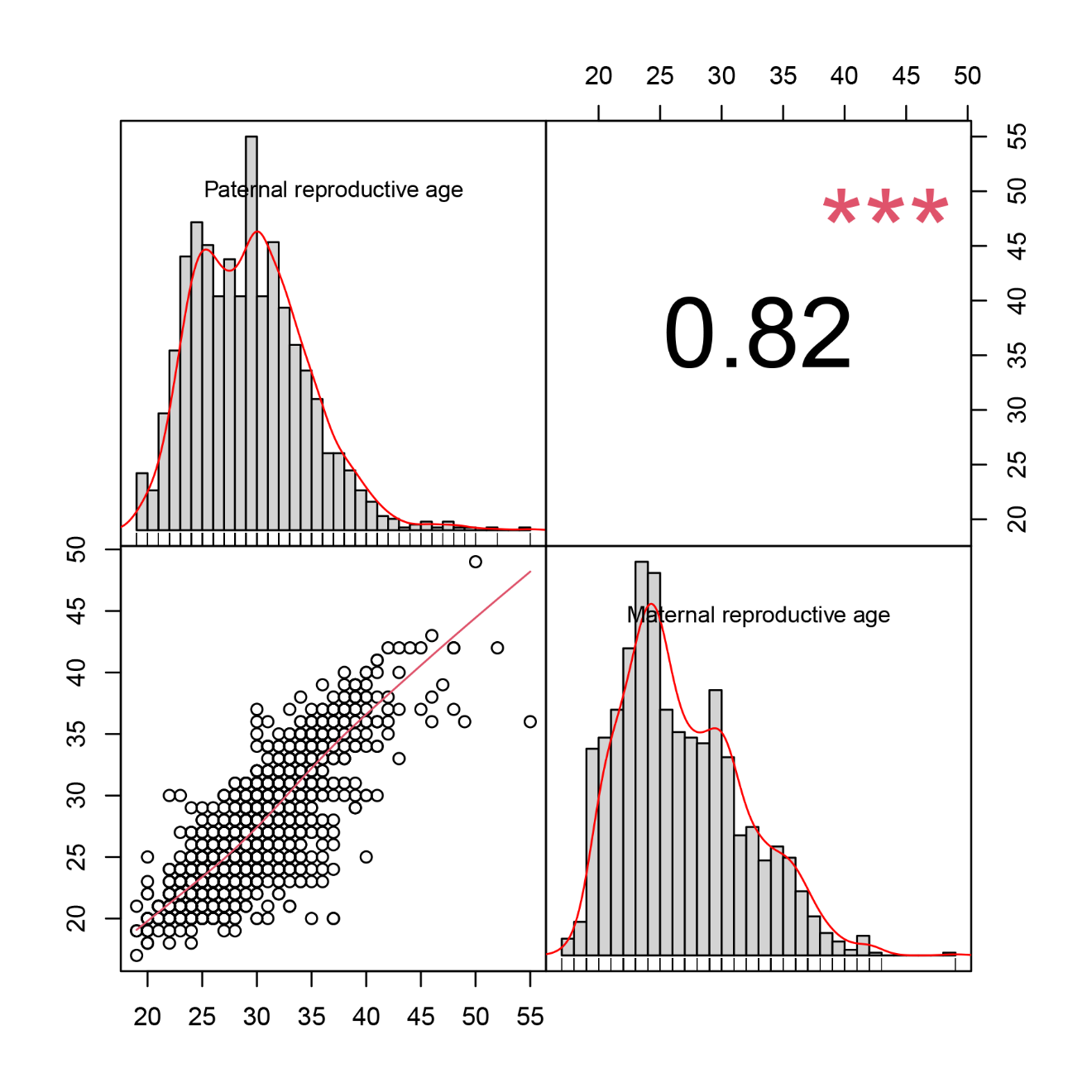


**Supplementary Figure S1** Correlation between paternal and maternal age at childbirth.

Note: Pearson correlation analysis was used. The left diagonal indicates histograms of the distribution of paternal and maternal ages at childbirth. The lower left triangle depicts the related trends in paternal and maternal reproductive age. The values in the upper right triangle are the correlation coefficients, and *** indicates a p-value less than 0.001.

**
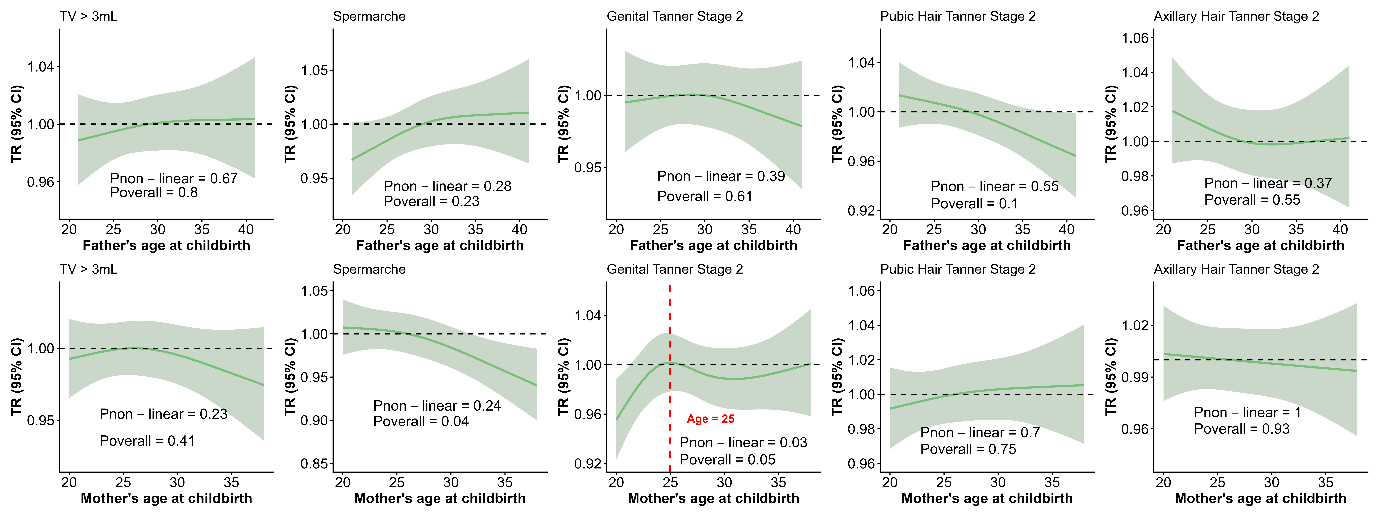
**

**Supplementary Figure S2** Association between parents' age at childbirth and the timing of puberty in boys (interpolated data).


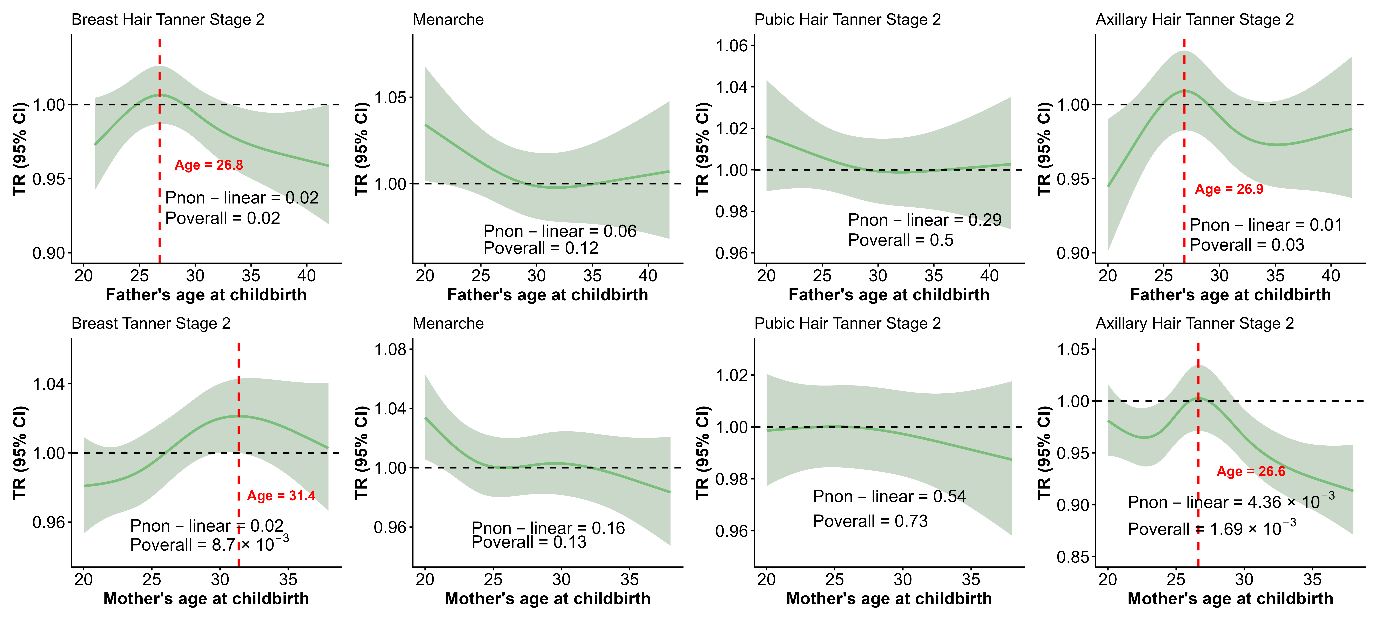


**Supplementary Figure S3** Association between parents' age at childbirth and the timing of puberty in girls (interpolated data).
